# Maintenance of a High Quality of Life of a Pancreatic Cancer Patient in a Multidisciplinary Care Center: A Case Report

**DOI:** 10.1155/crom/1775859

**Published:** 2025-11-18

**Authors:** Takayoshi Ubuka, Keiko Kato, Takeshi Hirose, Ken Sugimoto, Yoichi Yamane

**Affiliations:** ^1^Department of Healthcare, SI Research Institute, SI Holdings plc., Chuo-ku, Tokyo, Japan; ^2^First Department of Medicine, Hamamatsu University School of Medicine, Hamamatsu, Shizuoka, Japan; ^3^SI Research Institute, SI Holdings plc., HOSO-Kiko General Incorporated Association, Chuo-ku, Tokyo, Japan

**Keywords:** care center, chemotherapy, end-of-life care, pain management, pancreatic cancer, physical exercises, quality of life, social activities

## Abstract

**Introduction:**

The symptom and treatment of pancreatic ductal adenocarcinoma (PDA) at an advanced stage significantly deteriorate patients' quality of life (QOL). Therefore, multidisciplinary long-term and palliative care is needed to maintain patients' physical, social, and psychological well-being and ultimately improve survival.

**Case Presentation:**

An octogenarian residing in a care center, which can provide multidisciplinary long-term care with nutritional, physical, and social support, as well as pain management through home medical services, was diagnosed with Stage IV PDA. She received multiple outpatient chemotherapy sessions and experienced cancer-related pain and fatigue. However, participating in individual functional training and altruistic social activities at the day service center, which is a crucial component of Japan's long-term care system, within the care center maintained her high level of QOL for 5 years and 5 months until she died peacefully at the care center.

**Conclusion:**

Multidisciplinary care with nutritional support, palliative care, exercise, and social support at the care center may have reduced the patient's psychological stress and maintained her physical and psychological well-being and ultimately improved her QOL and survival. The multidisciplinary care center may be a candidate for a new model system of palliative and hospice care that did not only provide pain/symptom control and terminal care but also maintained a high QOL of the patient.

## 1. Introduction

Pancreatic ductal adenocarcinoma (PDA) presents at an advanced stage and causes symptoms that significantly deteriorate the patient's quality of life (QOL) [1]. Therefore, early implementation of multidisciplinary care with nutritional support, exercise, and palliative care is necessary to maintain patients' physical and psychological well-being and ultimately improve survival [1]. Qualitative and quantitative studies further highlighted the importance of social support in maintaining the physical and psychological well-being of individuals managing life-limiting illnesses [2, 3].

This case report presents a case of an octogenarian who was diagnosed with Stage IV PDA. The patient was a resident of a care center that is a multidisciplinary care platform with a residential area [4]. Individuals aged 60 and older who have been certified as requiring long-term care and their spouse can become residents of the care center. The residents can live a life with freedom and privacy like living in a normal apartment. In addition, the manager checks their safety every day, and the residents can consult anytime about their health and living. Purchase of welfare equipment and referral to visiting care and/or home medical care services are possible. The barrier-free residence room and bathroom also have a 24-h emergency call system. Residents can eat nutritionally balanced meals (breakfast, lunch, and dinner) in the dining room supervised by a registered dietitian upon request.

Most residents of the care center use the elderly day service center within the care center, which is open from 9 a.m. to 5 p.m. Outside users, who are not the residents, can also participate in the day service center. The day service center is a crucial component of Japan's long-term care system, which provides support for older persons who require assistance with daily living activities and aims to prevent further decline in their physical and cognitive functions [5]. In the morning, users of the day service center can take a bath with care after a health check by a nurse. Users usually engage in physical and oral exercises before having a nutritionally balanced lunch. Caregivers also offer various recreational activities, individual functional training, and activities such as cognitive training and occupational and reminiscence therapies. Socializing with other users in the pseudosociety including normal local older citizens may also play a significant role in maintaining the users' physical and mental health [5].

The care levels to be supported by the long-term care insurance system are decided by the municipal officials based on the investigation official's and home doctor's opinions. The care manager helps choose the service and writes the care plan that determines the overall policy. Actual daily care, such as bathing, meals, individual functional training, and recreational activities, is provided by the care staff at the day service centers according to the care plan. At least one full-time nursing staff must be available at the day service center to manage the health of the users, provide basic medical care, support care staff, and respond to emergencies. They perform medical procedures such as checking vital signs, managing medication, and deciding whether users can bathe or apply ointments. Advanced and emergency medical care is provided by hospitals or home-visit doctors and nurses, which are based on the medical insurance system. Regular attendance of a certified social worker who listens to the consultations from the users and their families and supports them in resolving concerns and issues in their daily lives is mandatory at the day service center. The role of the certified social worker includes coordinating with various professionals such as care managers and caregivers to ensure that the most appropriate care services are provided.

## 2. Case Report/Case Presentation

### 2.1. Patient's Information

An octogenarian resident of the care center had a history of uterine cancer in her 40s and bladder cancer in her 60s. Both cancers were believed to have been fully eradicated through surgical resection and radiation therapy. Her medical history also included adhesive ileus, spinal canal stenosis, and secondary lymphedema.

Institutional review board approval was obtained for case reporting. Written informed consent for publication was provided by the family member who was legally authorized to provide consent on behalf of the deceased pancreatic cancer patient.

### 2.2. Diagnostic Assessment and Therapeutic Intervention

A mass exhibiting inadequate contrast enhancement was detected in the body of the pancreas through computed tomography (CT) to monitor bladder cancer treatment (Table 1, 0Y0M). The tumor was deemed unresectable because CT revealed marked dilation of the distal pancreatic duct, invasion of the distal celiac, left gastric, splenic, common hepatic arteries, and retroperitoneal tissue. A tumor measuring 41 mm in diameter was observed in the pancreatic body by abdominal contrast-enhanced CT. The invasion was close to the superior mesenteric artery trunk 1 month later (0Y1M). The pancreatic body cancer was therefore diagnosed as Stage IV PDA.

The first course of gemcitabine and nab–paclitaxel combined therapy (GEM + nab-PTX) was started (0Y1M). Tegafur/gimeracil/oteracil (S-1) was also started 2 months later (0Y3M). The tumor size decreased to 20 mm on CT. However, arterial encasement was still observed (0Y4M). S-1 was started again (0Y5M). Increases in right pleural effusion and lung-field density were observed by CT (0Y6M). During this time, she was hospitalized for 12 days due to intestinal obstruction (0Y7M to 0Y8M). She was hospitalized for 5 days due to a urinary tract infection (1Y4M). Although significant changes were not observed in the primary tumor, a slight increase in ascites was observed by CT (1Y7M). During this time, she was rehospitalized for 9 days due to intestinal obstruction (1Y7M).

S-1 was started (1Y8M), followed by the tenth course of GEM + nab-PTX (1Y9M). She was rehospitalized for 9 days due to intestinal obstruction (1Y11M). CT did not detect significant changes in the primary tumor (1Y11M, 2Y3M). S-1 was restarted (2Y4M, 2Y5M). CT revealed increased soft tissue densities in the lower pancreatic region, suggesting disease worsening (2Y6M), and GEM + nab-PTX was resumed (2Y7M). Despite decreases in soft tissue density in the distal part of the pancreatic body and left para-aortic area, the density above the nodule in the left lower lobe increased based on CT findings (2Y12M). CT detected localized tumor growth and invasion around the proper hepatic artery (3Y4M). Combined treatment with irinotecan + fluorouracil (5-FU) + levofolinate (LV) was started (3Y7M).

**Figure 1:**
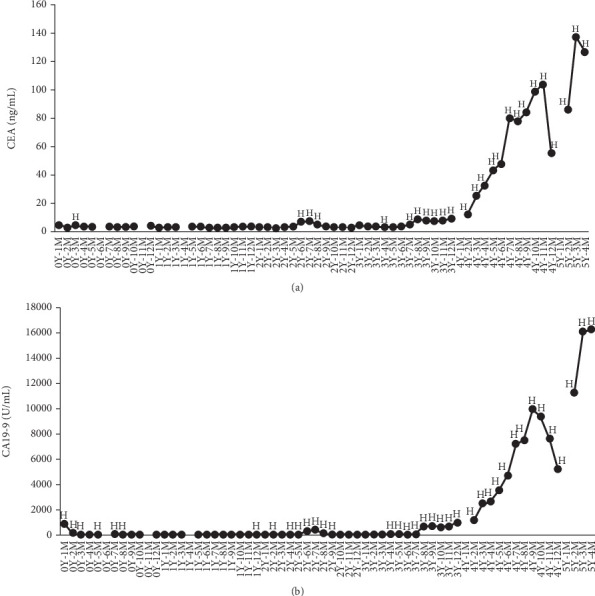
Plasma concentrations of carcinoembryonic antigen (CEA) and carbohydrate antigen 19-9 (CA19-9). H indicates higher concentrations beyond the reference values of (a) CEA (5.0 ng/mL) and (b) CA19-9 (37 U/mL). The year (Y) and month (M) indicate the time after the initial diagnosis of pancreatic cancer. Data 6 months (0Y6M), 11 months (0Y11M), 1 year and 4 months (1Y4M), 4 years and 1 month (4Y1M), and 5 years and 2 months (5Y2M) after the initial diagnosis of pancreatic cancer were not available.

The possibility of metastasis to the lung was observed (3Y12M). She was admitted to the hospital for 10 days due to respiratory distress (3Y12M). Ascites was increased (4Y2M), and the possibility of peritoneal dissemination was observed (4Y6M). After the last course of irinotecan + 5-FU + LV (4Y8M), GEM + nab-PTX was resumed (4Y9M to 4Y12M). During this time, she was hospitalized for 21 days due to worsening pleural effusion (4Y12M to 5Y1M). Circulating carcinoembryonic antigen (CEA) and carbohydrate antigen 19-9 (CA19-9) levels were regularly quantified to monitor the inhibition and growth of the pancreatic cancer (Figure 1).

### 2.3. Pain Management

The patient had been taking oxycodone at the care center by the home medical service from 3Y11M. Hydromorphone was additionally used to control cancer-related pain from 5Y4M.

### 2.4. Participations in the Activities of the Day Service Center and the Patient's QOL

**Figure 2:**
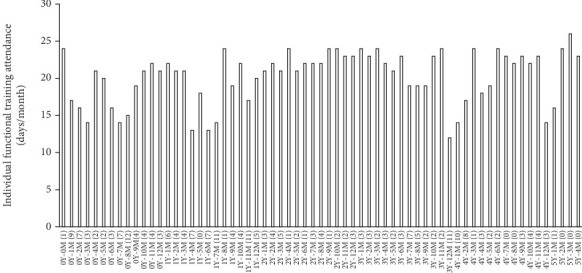
Participation frequencies of individual functional training of the resident. Participants usually take part in group training, whereas individual functional trainings are designed for each participant. The year (Y) and month (M) indicate the time after the initial diagnosis of pancreatic cancer. The numbers in parentheses after the year and month indicate the total number of days that the resident of the care center was unable to participate in the activities of the day service center due to outpatient visits or hospitalization.

She regularly participated in the activities of the day service center, except on the days she had to go to the hospital as an outpatient or when she was hospitalized. She usually participated in individual functional training assisted by a nurse or caregiver at the day service center (Figure 2). The morning program consisted of heel raising, toe raising, knee extensions, finger exercises, adductor muscle training, abductor muscle training, iliopsoas muscle training, leg raising, wide stride walking, ball exercises, TheraBand training, and rhythmic gymnastics that took approximately 30 min. She participated in individual functional training until 10 days before her death (Figure 2, 5Y4M).

**Figure 3:**
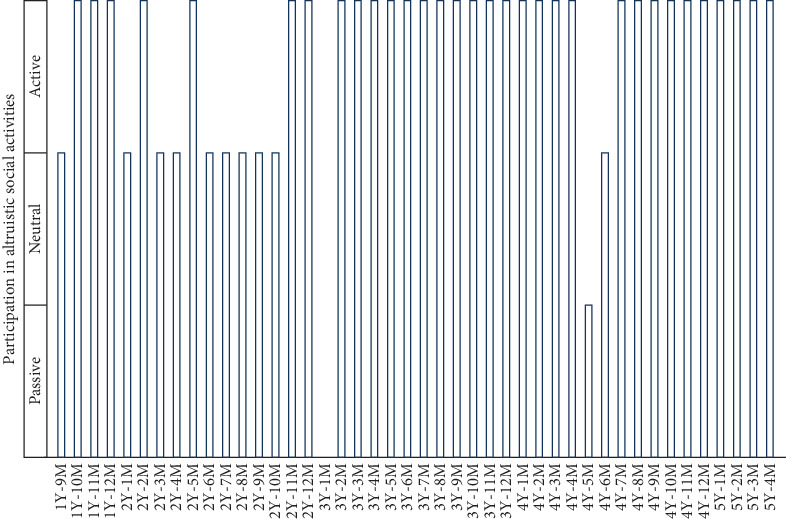
Participation in altruistic social activities. Activities consisted of occupational activities such as cooking and hanging and folding the laundry. The attitude of her participation in altruistic social activities (active, neutral, and passive) in each month was qualified by the certified caregiver. The year (Y) and month (M) indicate the time after the initial diagnosis of pancreatic cancer. No data were available 3 years and 1 month (3Y1M) after the initial diagnosis of pancreatic cancer.

She also enjoyed cognitive training, physical exercises, and various recreational activities, such as making crafts and drawing pictures. She was especially happy to participate in occupational activities such as cooking and hanging and folding the laundry. She always helped other users when they had difficulties in their tasks. She often listened to other users' concerns and was always a good advisor. She was active in participating in these altruistic social activities until her last month (Figure 3, 5Y4M). The altruistic social activities gave her a purpose in life and improved her QOL.

**Figure 4:**
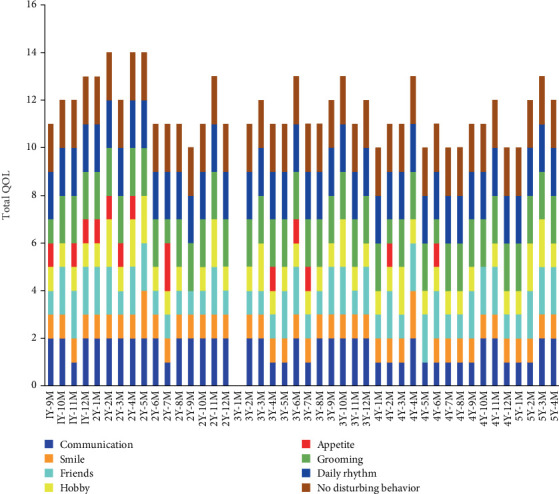
Quality of life (QOL) of the resident. The year (Y) and month (M) indicate the time after the initial diagnosis of pancreatic cancer. No data were available 3 years and 1 month (3Y1M) after the initial diagnosis of pancreatic cancer.

Degrees of communication, smile, friends, hobby, appetite, grooming, daily rhythm, and absence of disturbing behavior were summarized each month by the certified social worker based on the observations by the caregivers and nurse in charge. The three-stage evaluation criteria (2, 1, and 0 points) were as follows: communication (2, *positive and spontaneous communication*; 1, *passive communication*; 0, *few communication*), smile (2, *always smiling*; 1, *seen smiling*; 0, *smiling a few times*), friends (2, *has several close friends*; 1, *has a close friend*; 0, *no close friend*), hobby or purpose in life (2, *living a purposeful life*; 1, *feeling joy and happiness*; 0, *no joy and happiness*), appetite (2, *eats it all*; 1, *eats approximately 80%*; 0, *no appetite*), grooming (2, *well-groomed*; 1, *concerned about appearance*; 0, *not concerned about appearance*), daily rhythm (2, *living a regular life*; 1, *life rhythm tends to be disrupted*; 0, *daily rhythm is disrupted*, day–night reversal), and absence of disturbing behavior (2, *no disturbing behavior*; 1, *occasional disturbing behavior*; 0, *frequent disturbing behavior*). The patient's total QOL, measured by the sum of communication, smile, friends, hobby, appetite, grooming, daily rhythm, and absence of disturbing behaviors, was high even after disease worsening (Figure 4). However, her appetite was generally diminished, and she occasionally experienced a lack of joy and happiness during the 2 years and 9 months (2Y9M) and 4 years and 10 months (4Y10M) following the initial diagnosis of pancreatic cancer (Figure 4).

### 2.5. Advance Care Planning (ACP) and End-of-Life Care

The ACP meeting was held with the medical and long-term care support team that consists of a home-visit doctor and nurse, the director of the day service center, and the patient and her family 4Y10M after the initial diagnosis of pancreatic cancer (4Y10M). According to the confirmation of intention document filled and signed after the ACP meeting, she did not wish to be transported to the hospital and receive intensive testing and medical care when her health deteriorated. Furthermore, she did not wish to be taken to the hospital by emergency services and receive life-prolonging treatments such as cardiopulmonary resuscitation, preferring instead end-of-life care at the care center.

Home-visit nursing care service was started at her residence in the care center every morning and evening, before and after she spends time at the day service center, from the day after she was discharged from the hospital following 21 days of admission due to worsening pleural effusion (5Y1M). The visiting nurse regularly checked her body temperature, pulse, blood pressure, and oxygen saturation (SpO_2_) and performed nursing care such as medication management. The home-visit doctor prescribed oxygen inhalation after her SpO_2_ levels dropped to < 90%, and for 2 weeks, she received 1 L of oxygen daily until she began experiencing respiratory distress (5Y4M). At 6:51 a.m., she made an emergency call reporting difficulty in breathing, prompting an emergency visit from the home-visit nurse. At this time, her oxygen supply was increased to 2 L, which led to an improvement in her respiratory distress. However, her SpO_2_ level rose only to the 80% range. The following morning, her SpO_2_ dropped < 80%, necessitating an increase in oxygen supply to 3 L. Unfortunately, her SpO_2_ became undetectable, and she died peacefully at the care center 5 years and 5 months (5Y5M) after the initial diagnosis of pancreatic cancer.

## 3. Discussion

The pancreatic body cancer was unresectable in this case due to distant metastasis and the patient was diagnosed as Stage IV PDA shortly after its initial observation by CT. GEM + nab-PTX therapy was started without delay as the first-line treatment recommended by the Japanese Clinical Practice Guidelines for Pancreatic Cancer 2019 [6]. A Phase III study demonstrated the survival benefits of GEM +nab-PTX therapy compared with GEM monotherapy [7]. The median overall survival was 8.5 months in the GEM + nab-PTX group, as compared with 6.7 months in the GEM group. The median progression-free survival was 5.5 months in the GEM + nab-PTX group, as compared with 3.7 months in the GEM group. The survival rate was 35% in the GEM + nab-PTX group versus 22% in the GEM group at 1 year and 9% versus 4% at 2 years [7]. Even considering that S-1 was additionally used during the GEM + nab-PTX therapy [8], and irinotecan + 5-FU + LV was used as the second-line treatment [6]. 5Y5M' survival and the maintenance of a high QOL over 5 years after the initial diagnosis of Stage IV PDA suggest the benefit of integrating multidisciplinary care, social support, and community-based services in managing advanced cancer.

Pancreatic cancer is characterized by aggressive tumor growth with early metastasis. Cancer cachexia and sarcopenia are ubiquitous characteristics that limit daily activities, compromise patients' QOL, and are associated with poor overall survival [9]. Therefore, preservation of physical functioning and QOL becomes the main goals of supportive cancer care. Physical exercises were shown to have positive effects on the QOL of pancreatic cancer patients in randomized trials [10–12]. The patient in this case was diagnosed with Stage IV PDA and received multiple chemotherapy sessions and suffered from cancer-related pain and fatigue. However, as a resident of the care center, she was able to eat nutritionally balanced meals, and cancer-related pain was controlled by the home-visit medical team at the care center [13]. She was thus able to regularly engage in personalized functional exercise that may have contributed to maintaining her QOL.

Social isolation was reported to be associated with low well-being and worsening depression [14], severe pain [15], and mortality [16]. QOL was compared among patients with cancer treated at home, at an in-patient palliative care unit, and at a day care center [17]. The European Organization for Research and Treatment of Cancer QOL Questionnaire Core-15-Palliative Care, Edmonton Symptom Assessment System (ESAS), and Karnofsky Performance Status (KPS) scale identified that patients treated at the day care center had the highest QOL scores [17]. Conversely, the ESAS and KPS scales identified that QOL deteriorated in patients treated at home and at an in-patient palliative care unit, suggesting the negative effect of social isolation on the QOL of patients with cancer [17]. The positive effects of specialist palliative day care on the QOL of patients with and without cancer in an environment that allows patients to socialize with peers through various recreational activities were also reported [18]. The day service center provided opportunities for social interaction with normal local senior citizens including nonterminally ill patients, which contributed to maintaining the QOL of the patient. Especially, altruistic social activities within the pseudosociety of the day service center gave the patient a purpose in life and significantly improved her QOL.

Depression and anxiety were reported to be associated with an increased risk of cancer-specific and all-cause mortality [19]. The negative effects of depression and anxiety on cancer progression may be at least partially related to the negative effects of psychological stress in immune dysfunction that influence tumor behaviors [20]. Therefore, various stress interventions, including physical exercises and psychological relaxation, have been administered to reduce mental disorders and improve the QOL of patients with cancer [21]. Early implementation of multidisciplinary care with nutritional support, palliative care, exercise, and social support at the care center may have reduced her psychological stress and maintained her physical and psychological well-being and ultimately improved her QOL and survival.

Although home hospice services are common in the United States, most of the hospice services in Japan are provided by the hospital [22]. Although the hospice service in the United States includes nursing care, medical social services, physician services, consultation, home care aid, homemaker services, supply of medical equipment, and physical and occupational therapies based on the hospice act designated in 1981, the hospice service in Japan provides only pain/symptom control and minimum physical, psychological, and terminal care for terminal AIDS and cancer patients within the closed environment of the hospital [22]. The multidisciplinary care center of this case can be a new candidate system of palliative and hospice care that not only provides pain/symptom control and terminal care but also maintains the patient's physical and psychological well-being and survival.

## Figures and Tables

**Table 1:** Chemotherapy, inhibition, growth, and possible metastasis of cancer.

**Y**	**M**	**Chemotherapy**	**Cancer condition**
0	0		A mass with poor contrast was found in the body of the pancreas by CT.
	1		Pathological examination of the EUS-FNA sample was false positive because the number of abnormal pancreatic cancer-like cells was small. However, the tumor was diagnosed to be unresectable because marked dilation of the distal pancreatic duct, invasion to splenic artery, common hepatic artery, and retroperitoneal tissue was observed by CT.
	1		A tumor of a 41-mm-long diameter was observed in the pancreatic body by abdominal contrast-enhanced CT. Invasion to distal celiac, left gastric, splenic, and common hepatic arteries was also observed. Invasion was close to the superior mesenteric artery trunk.
	1	The first course of GEM + nab-PTX was started.	
	3	S-1 was started.	
	4		The decrease in the tumor size to 20 mm was observed by abdominal contrast-enhanced CT. Arterial encasement remains.
	5	S-1 was started.	
	6		Increases in the right pleural effusion and the lung field density were observed by CT.
	10		The tumor size was not changed as observed by abdominal contrast-enhanced CT.

1	7		Although significant changes were not observed in the primary tumor, slight increase in ascites was observed by CT.
	8	S-1 was started.	
	9	The tenth course of GEM + nab-PTX was started.	
	11		Significant changes were not observed in the primary tumor by CT.

2	3		Significant changes were not observed in the primary tumor by CT.
	4	S-1 was started.	
	5	S-1 was started.	
	6		Increase in various soft tissue densities was observed in the lower pancreatic region by abdominal contrast-enhanced CT, suggesting a worsening of the disease.
	7	GEM + nab-PTX was resumed.	
	12		Although decreases in soft tissue density in the distal part of the pancreatic body and the left para-aortic area was observed, increased density above the nodule in the left lower lobe was observed by abdominal contrast-enhanced CT.

3	4		Localized tumor growth and invasion around the proper hepatic artery were observed by abdominal contrast-enhanced CT.
	7	Irinotecan + 5-FU + LV was started.	
	12		Possibility of metastasis to the lungs.

4	2		Increased ascites.
	6		Possibility of peritoneal dissemination.
	8	The last course of irinotecan + 5-FU + LV.	
	9	GEM + nab-PTX was resumed.	
	12	The last course of GEM + nab-PTX.	

Abbreviations: 5-FU, fluorouracil; CT, computed tomography; EUS-FNA, endoscopic ultrasound–fine needle aspiration; GEM + nab-PTX, gemcitabine and nab–paclitaxel combined therapy; LV, levofolinate; M, month; S-1, tegafur/gimeracil/oteracil; Y, year.

## Data Availability

The data that support the findings of this study are not publicly available due to privacy reasons but are available from the corresponding author upon reasonable request.
